# Quality of life among people living with mental illness and predictors in Africa: a systematic review and meta-analysis

**DOI:** 10.1007/s11136-023-03525-8

**Published:** 2023-10-31

**Authors:** Wondale Getinet Alemu, Clemence Due, Eimear Muir-Cochrane, Lillian Mwanri, Telake Azale, Anna Ziersch

**Affiliations:** 1 College of Medicine and Public Health, Flinders University Adelaide, Adelaide, Australia; 2 Department of Psychiatry, College of Medicine and Health Sciences, University of Gondar, Gondar, Ethiopia; 3 School of Psychology, The University of Adelaide, Adelaide, Australia; 4 College of Nursing and Health Sciences, Flinders University, Adelaide, Australia; 5 Research Centre for Public Health, Equity and Human Flourishing, Torrens University Australia, Adelaide Campus, Adelaide, SA Australia; 6 Institute of Public Health, College of Medicine and Health Sciences, University of Gondar, Gondar, Ethiopia

**Keywords:** Mental illness, Mental disorder, Quality of life, Mental wellbeing, Africa

## Abstract

**Introduction:**

Quality of life (QoL) of patients with mental illness has been examined internationally but to a lesser extent in developing countries, including countries in Africa. Improving QoL is vital to reducing disability among people with mental illness. Therefore, this systematic review and meta-analysis aimed to assess the prevalence of QoL and associated factors among people living with mental illness in Africa.

**Methods:**

Using the PICOT approach, Scopus, MEDLINE, PsycINFO, CINAHL, Embase, the Web of Science, and Google Scholar were searched. A structured search was undertaken, comprising terms associated with mental health, mental illness, QoL, and a list of all African countries. The Joanna Briggs Institute Quality Appraisal Checklist is used to evaluate research quality. Subgroup analysis with Country, domains of QoL, and diagnosis was tested using a random-effect model, and bias was assessed using a funnel plot and an inspection of Egger's regression test. A *p* value, OR, and 95% CI were used to demonstrate an association.

**Results:**

The pooled prevalence of poor QoL was 45.93% (36.04%, 55.83%), *I*^2^ = 98.6%, *p* < 0.001). Subgroup analysis showed that Ethiopia (48.09%; 95% CI = 33.73, 62.44), Egypt (43.51%; 95% CI = 21.84, 65.18), and Nigeria (43.49%; 95% CI = 12.25, 74.74) had the highest mean poor QoL prevalence of the countries. The pooled prevalence of poor QoL by diagnosis was as follows: bipolar disorder (69.63%; 95% CI = 47.48, 91.77), Schizophrenia (48.53%; 95% CI = 29.97, 67.11), group of mental illnesses (40.32%; 95% CI = 23.98, 56.66), and depressive disorders (38.90%; 95% CI = 22.98, 54.81). Being illiterate (3.63; 95% CI = 2.35, 4.91), having a comorbid medical illness (4.7; 95% CI = 2.75, 6.66), having a low monthly income (3.62; 95% CI = 1.96, 5.27), having positive symptoms (0.32; 95% CI = 0.19, 0.55), and having negative symptoms (0.26; 95% CI = 0.16, 0.43) were predictors of QoL. Thus, some factors are significantly associated with pooled effect estimates of QoL.

**Conclusions:**

The current systematic review and meta-analysis showed that almost half of patients with mental illness had poor QoL. Being illiterate, having a comorbid medical condition, having a low monthly income, having positive symptoms, and having negative symptoms of mental illness were independent predictors of poor QoL. This systematic review and meta-analysis emphasize that poor QoL of people with mental illness in Africa needs attention to reduce its negative consequences.

## Introduction

“Mental illness” refers to various mental health problems characterized by cognitive, behavioural, or emotional impairments significantly affecting functionality, essential living activities, and QoL for persons with the disorder [1]. A person's overall pleasure with their life and the belief that they are living the life they want is regarded as the QoL [2]. It relates to physical, emotional, and social wellbeing, emphasizing the patient's subjective assessment of their satisfaction with life [3, 4]. Patients with mental illnesses may need help to accurately evaluate their overall QoL due to symptoms and poor knowledge of the disease [5]. QoL can also be measured objectively to include indicators such as income, education, or health status, depending on the perspective and purpose of the assessment [6–9]. This review focuses on subjective QoL, drawing on the generic tools available to assess subjective QoL, including those developed by the World Health Organisation (WHO) [10–12].

Knowing the prevalence of mental disorders is a significant public health issue. For example, in a scoping review from twelve African countries, the lifetime prevalence of mood disorders ranged from 3 to 10%, anxiety disorders from 6 to 16%, substance use disorders from 4 to 13%, and psychotic disorders from 1 to 4% [13]. Pre-pandemic estimates placed the number of people in the African continent who were living with mental health disorders at over 116 million [14], 20.49% Rwandans have at least one mental [15], 12.0% Nigeria [16] and 30.3% South Africa [17], with significant variations across the continent. Some European studies have also shown that geographical variations in the prevalence of mental disorders [18–20], cross-cultural differences in understanding of mental health, and measurement and diagnosis variations could account for variations in mental health prevalence rates [21].

While mental health care systems vary across African countries (and internationally), in most countries, there is a significant unmet need for those living with mental illness [22, 23], with mental health services under-staffed and poorly funded in most African countries [24]. At the policy level, few countries have mental health strategies [25], and even when they do have these, they may not be implemented in practice [26]. These situations leave those living with mental illnesses in many African countries forced to choose between living with untreated mental disease and/or seeking treatment from traditional or religious authorities due to the lack of services or the unaffordable high cost of care [27], impacting their QoL significantly [28].

QoL is a new concept that has received attention in the last two decades [29]. However, QoL is becoming more widely known in mental health studies [30, 31], which consider overall functionality and QoL [32]. Current evidence shows that the QoL of people with mental illnesses is highly impaired in developed and developing countries [30], including Africa where limited research is available. Internationally, prevalence studies provide evidence that patients with severe mental illnesses have lower QoL than the general population. For example, in Europe, poor QoL was found among 34% of patients with mental illness in Germany, which is a lower rate than the general population. In Spain, 31% of individuals with unipolar depression were reported to have poor QoL [33]. In Ethiopia, the prevalence of poor QoL scores of patients with severe mental illness was reported to be 41%, 43%, 39%, and 42% for the physical, psychological, social, and environmental domains, respectively [34]. Variations in the QoL in patients with mental illness across countries are likely to reflect a range of factors, including cultural understandings of mental illness and health and support systems.

Previous studies have highlighted sociodemographic and clinical variables as important determinants of QoL [29] for major mental illnesses, including depression [35, 36] and Schizophrenia [37, 38]. For example, a recent study with schizophrenic outpatients in Jordan found that QoL was negatively correlated with the advanced age of the patients, male gender, longer duration of illness, high body mass index, and prescribed typical antipsychotic medication [39]. For patients with depression, the age of patients, age of onset of illness, sex, marital status, living, and sociodemographic factors were associated with variation in QoL [40, 41]. QoL for people with mental illness has been positively correlated with social support [42], medication adherence [43], and co-occurring disorders [33, 44–50]. As for QoL itself, likely, the impact of these factors on QoL for people with mental illness may vary between countries depending on the cultural context and health system and other variations.

The systematic and multi-dimensional use of the QoL concept has been recommended for planning therapies, monitoring outcomes in studies, and patient management [51, 52]. Recently, researchers have shown an increased interest in evaluating QoL and determinant factors because QoL is increasingly used as an outcome indicator for mental disorders [29, 37]. However, to our knowledge, there has been no previous systematic review or meta-analysis of the QoL and its determinants in Africa. Thus, the purpose of the current systematic review and meta-analysis was to investigate the QoL of patients with mental illness in Africa and significant predictors. This paper reviews the data from previous research, aiming to answer the following two questions: 1. What is the pooled prevalence of QoL among people with mental illness in Africa? 2. What are the predictors of QoL among people with mental illness in Africa?

## Methods

### Reporting and protocol registration

This systematic review and meta-analysis protocol was registered on the International Prospective Register of Systematic Reviews (PROSPERO) with CRD 42022333309[53]**.** Respectively, the selection of articles and reporting review results have employed the Preferred Reporting Items for Systematic Reviews and Meta-Analysis (PRISMA) criteria and flow diagram [54]. The findings are reported according to the study area (Country), the type of mental illness, and any relevant variables.

### Search strategies

We conducted electronic and manual searches to identify publications for the systematic review and meta-analysis. Electronic data were accessed by searching Scopus, MEDLINE, PsycINFO, CINAHL, Embase, the Web of Science, and Google Scholar. With the assistance of a qualified Flinders University research librarian, a search strategy was created. The PICOT technique (Eriksen and Frandsen, 2018) [55] was used to develop the systematic search strategy described as follows: The P (population of interest) consisted of Africans who were suffering from mental illness. There were no interventions (I), controls (C), or comparison groups needed for this study; QoL was the outcome (O). Peer-reviewed and grey literature on the subject that discussed the outcome of interest were identified. All empirical research studies that published primary quantitative data relevant to the study themes were examined (T) by type of study. The full search strategy can be seen at Supplementary File. The search was conducted on 23/10/2022.

## Eligibility criteria

### Types of studies, types of participants, outcomes, and context

Peer-reviewed publications that reported the prevalence and/or predictors of QoL of adults with any type of mental illness conducted in Africa as cross-sectional, case–control, and cohort studies were considered for inclusion. Studies published in English conducted in hospitals or outpatient clinics with outcomes of interest were included for review if they showed the prevalence of QoL or its determinants.

### Screening process

Research articles obtained from the specified databases were imported into EndNote X20 before being transferred to Covidence. Covidence was used to remove duplicates of full-text articles and abstracts. Full texts were read after screening titles and abstracts. When studies were found in databases but did not have complete information, the corresponding authors were contacted via email to get adequate information.

### Methodological quality of articles

Before inclusion, relevant papers were assessed for methodological Quality. The authors (WGA and EMC) separately evaluated the risk of bias in each article, which was designed to determine the Quality of prevalence studies and the methodological Quality of the papers using the Joanna Briggs Institute Critical Appraisal checklist [56]. Nine items make up the JBI checklist. Each question received a score (0 for “not reported” or “not appropriate” and 1 for “yes”); the item scores were added to provide a total quality score ranging from 0 to 9. Studies whose scores were between 0 and 4, 5 and 7, and 8 or 9 were then classified as low, moderate, or high Quality, respectively. The final analysis includes studies with high or moderate Quality. There were no discrepancies in the methodological quality assessment between evaluators on articles included.

### Data extraction

After appropriate studies were found, a Microsoft Excel spreadsheet with a pre-formatted layout was used for data extraction. The following parameters were used to extract the variables: author(s), sample size and response rate, year of publication, study area (Country), and participant characteristics (diagnosis).

### Data analysis

We computed the logarithm of prevalence and the standard error of the logarithm of prevalence to obtain a pooled prevalence of QoL in people with mental illness and pooled effect estimates of predictor variables. STATA 17 was used for analysis, and statistical meta-analysis was conducted to analyse the collected data and determine the pooled prevalence of QoL among people with mental illness in Africa. First, a random-effect model was used to show summary statistics, and heterogeneity across the studies was examined using the *I*^2^ heterogeneity test and Cochran’s *Q* test [57]. The *I*^2^ heterogeneity was determined as the 25%, 50%, and 75% thresholds used to indicate low, moderate, and severe heterogeneity, respectively [58, 59]. A subgroup analysis was performed considering the disorder diagnosis, study region, and tool used to measure the outcome. The bias of the small study was examined through a funnel plot and an objective inspection of Egger's regression test [60]; Publication bias was declared if the shape of the funnel plot was asymmetrical or if Egger's regression assumption test result was statistically significant (*p* < 0.05)[61, 62]. The pooled prevalence and the pooled effects estimate of the odds ratio were presented at a 95% CI.

## Results

### Search results

**Fig. 1:**
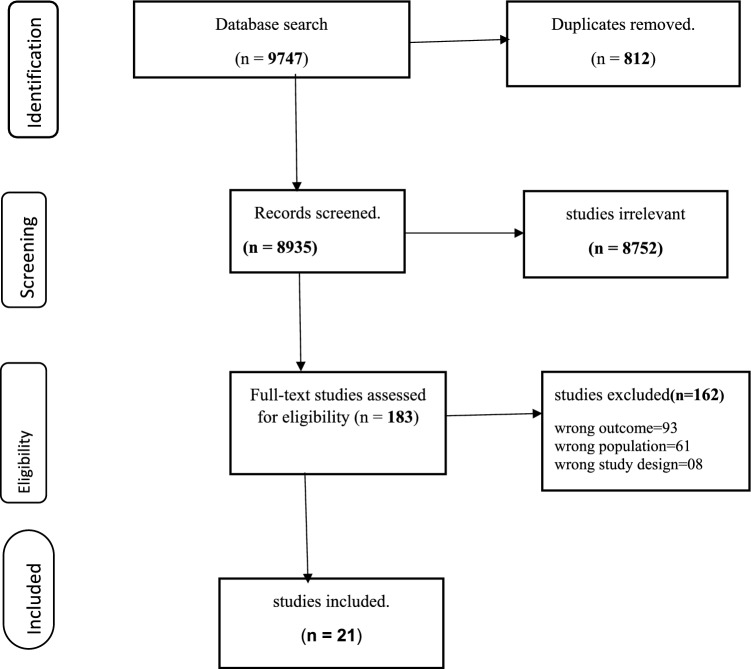
Prisma diagram shows the selection of studies for the systematic review and meta-analysis of prevalence and associated factors of QoL among people with mental illness in Africa.

The procedures for screening and excluding articles were shown using a PRISMA flow diagram (Fig. 1).

Guidelines of the Meta-analysis of Observational Studies in Epidemiology [63] and the PRISMA checklist were applied in reporting the study results [54]. A total of 9747 articles were found in the databases used. Among the available total, 812 articles were duplicates, 8935 records were screened, and 8752 were assessed as irrelevant after screening the titles and abstracts, and they were excluded from the analysis. Furthermore, of 183 full-text studies evaluated for eligibility, 162 articles were ineligible for reasons including results of wrong outcome (*n* = 93), wrong population (*n* = 61), and wrong study design (*n* = 8). A total of 21 articles were assessed as eligible and were included for analysis (Fig. 1).

### Overall characteristics of articles

All the 21 full-text articles included in the current study were cross-sectional studies. The total number of respondents in each study ranged from 70 in a study conducted in Egypt [71] to 487 in a study conducted in Nigeria [79]. A total of 5665 study participants participated across the studies. The included articles were conducted in seven different African countries and were published between 2005 [82] and 2021 [76]. Eight studies were conducted in Ethiopia [64–70, 81], five studies in Egypt [71–74, 83], four studies in Nigeria [77–79, 84], and there was one study each in Kenya [75], Uganda [76], South Africa, [80] and Sudan, [82] respectively. The response rate of the included studies ranged from 97% [70] to 100% [65]. Fifteen studies used the WHOQOL-Brief to measure the QoL, two used the SF-36, one EUROHIS, two studies used the Quality of life index, and one with schizophrenia QoL scale (Table 1).

**Table 1:** Descriptive summary of the prevalence and associated factors of Quality of life among people with mental illness in Africa (*n* = 21)

No	Authors and year	Sample characteristics & Country	Assessment method	Prevalence	Associated factors
1	Alem E, 2016 [64]	MI (Mental Illness,) *n* = 317 Ethiopia	SF-36 Cut off < / = mean	Poor QoL = 54.6% Physical = 53.1% Emotional = 45.6% Pain experience = 36.7% Social = 55.8% Energy = 61.7% Social life = 50.7% General health = 51.6%	Stigma (OR = 0.041; 95% C I: − 0.065, − 0.012)
2	Defaru D, 2018 [65]	Schizophrenia *n* = 352 Ethiopia	WHOQOL-26 Cut off < / = mean	Poor QoL = 74.34% Physical = 20.5% Psychological = 18% Social = 7.5% Environmental = 22.4%	Positive symptoms (β = − 0.33,95% C I: − 0.49 − 0.17) Negative symptoms (β = − 0.26,95% CI = − 0.45 − 0.06) General psychopathology (β = − 0.22,95% CI = − 0.32 − 0.12) Comorbid physical illness (β = − 4.69,95% CI = − 8.50 − 0.88) Khat use disorder (β = − 3.95,95% CI = − 6.02 − 1.88) Tobacco uses disorder (β = − 3.15,95% CI = − 5.34 − 0.95) medication nonadherence (β = − 5.82,95% CI = − 8.24 − 3.41)
3	Seid S, 2018 [66]	Depression *n* = 394 Ethiopia	WHOQOL-26 Cut off < / = mean	Poor QoL = 41.2% Physical = 41.3% Psychological = 42.8% Social = 38.9% Environmental = 41.8%	Age of respondents (physic, psychological, social, environmental domains) of QoL Age of onset of depression (physical and psychological domains) QoL Perceived stigma (physical domain) QoL Living arrangement (physical domain) QoL Poor social support (psychological, social, environmental) QoL Duration of illness (physical and environmental)
4	Tolesa F, 2018 [67]	Depression *n* = 418 Ethiopia	WHOQOL-26 Cut off < / = mean	Poor QoL = 13.6%	
5	Tolesa F, 2017 [68]	Schizophrenia *n* = 422 Ethiopia	WHOQOL-26 Cut off < / = mean	Poor QoL = 48.6%	Unable to read and write (OR = 4.42,95% CI = 1.40,14.08) Working in NGO (OR = 0.38,95% CI = 0.21, 0.70) Being on Risperidone (OR = 0.53,95% CI = 0.32, 0.87)) Having depression (OR = 3.77, 95% CI = 2.02, 7.05) Sexual dysfunction (OR = 4.90, 95% CI = 2.50, 9.59)
6	Tamrat A, 2020 [69]	Depression *n* = 423 Ethiopia	WHOQOL-26 Cut off < / = mean	Poor QoL = 47.5%	
7	Telake A, 2016 [70]	Bipolar disorder *n* = 423 Ethiopia	WHOQOL-26 Cut off < / = mean	Poor QoL = 58.4% Physical = 56.2% Psychological = 62.5% Social = 50.1% Environmental = 55%	Rural residence (OR = 1.94, 95% CI = 1.12–3.34) Primary education (OR = 3.08, 95% CI = 1.45–6.53) < 200 birrs monthly income (OR 3.57, 95% CI: 1.48–8.57) > 10 years duration of Illness (OR = 3.42, 95% CI = 1.19–9.77) 2-4episode of illness (OR = 3.45, 95% CI = 1.61–7.38) Antipsychotic & antidepressant medication (OR = 2.15, 95% CI = 1.04–4.45)
8	Soheir H, 2011 [71]	Schizophrenia *n* = 70 Egypt	QLI Cut off < / = mean	Poor QoL = 19.9%	Being a male *P* = 0.011 for the social domain Single (*P* = 0.009) Highly educated(*P* = 0.003) Unemployed or having a professional job (*P* = 0.001) Lower socioeconomic standard (*P* = 0.046)
9	Aya M, 2020 [72]	Schizophrenia *n* = 120 Egypt	SQLS < / = 75 Cut off < / = mean	Poor QoL = 55.8%	
10	Omnia M, 2012 [73]	MI *n* = 100 Egypt	QLI Cut off < / = mean	Poor QoL = 20%	
11	Hassan M, 2019 [74]	Depression *n* = 130 Egypt	WHOQOL-26 Cut off < / = mean	Poor QoL = 46% Physical = 58.5% Psychological = 73.1% Social = 46.2% Environmental = 42.3%	Family factors (*P* = 0.0001) Low social factors(*P* = 0.005) Educational level preparatory(*P* = 0.05) No sufficient income(*P* = 0.004)
12	Twahira S, 2017 [75]	Mental illness *n* = 374 Kenya	WHOQOL-26 Cut off < / = mean	Poor QoL = 19.5% Physical = 56.2% Psychological = 66.1% Social = 66.3% Environmental = 60.9%	Marital status (*P* = 0.003) Income (*P* = 0.011) Educational status (*P* = 0.441) Having work (*P* = 0.089)
13	Lucas A, 2021 [76]	Bipolar *n* = 169 Uganda	SF-36 Cut off < / = mean	Poor QoL = 81%	Having had suicidal thoughts (OR = 2.75, 95% CI = 1.14–6.63, *P* = 0.02) Psychotic symptoms (OR = 2.46, CI = 1.07–5.64, *P* = 0.03)
14	Temilola J, 2014 [77]	Schizophrenia *n* = 256 Nigeria	WHOQOL-26 Cut off < / = mean	Poor QoL = 81.25% Physical = 72.03% Psychological = 69.21% Social = 67.11% Environmental = 62.04%	Stigma(P = 0.003)
15	Adewuya AO 2006 [78]	Schizophrenia *n* = 99 Nigeria	WHOQOL-26 Cut off < / = mean	Poor QoL = 36.4% Physical = 22.08% Psychological = 21.94% Social = 7.77% Environmental = 22.6%	Anxiety/depression symptoms (OR = 4.88, 95% CI 2.93–11.48)) Comorbid medical problems (OR 4.75, 95% CI 1.43–16.33), Unemployment (OR 3.75, 95% CI 1.25–11.72) Poor social support (OR 4.60, 95% CI 1.49–14.28)
16	Omirin T, 2020 [79]	Mental illness *n* = 487 Nigeria	WHOQOL-26 Cut off < / = mean	Poor QoL = 45%	
17	Dumakazi M 2019 [80]	MI *n* = 121 South Africa	WHOQOL-26 Cut off < / = mean	Poor QoL = 27.3% Physical = 65.2% Psychological = 62.1% Social = 60.7% Environmental = 63.2%	Poor perceived social support Physical domain (*P* = 0.0064) Psychological domain (*P* = 0.0001) Social relationships domain (*P* = 0.0035) Environmental domain (*P* = 0.0006)
18	Yadeta A, 2019 [81]	Depression *n* = 415 Ethiopia	EUROHIS poor quality score level is < 24	poor QoL = 46.7%	
19	Awadalla AW, 2005 [82]	Schizophrenia *n* = 300 Sudan	WHOQOL-26 Cut off < / = mean	Poor QoL = 60%	
20	Amal S,2017 [83]	Mental illness *n* = 115 Egypt	WHOQOL-26 Cut off < / = mean	Poor QoL = 75.7% Physical = 59.1% Psychological = 67% Social = 80.9% Environmental = 67.8%	
21	Oluseun P, 2017 [84]	Schizophrenia *n* = 160 Nigeria	WHOQOL-26 Cut off < / = mean	Poor QoL = 11.2% Physical = 15% Psychological = 15.6% Social = 16.3% Environmental = 11.3%	

The term MI* – group of diagnosis of mental disorders

### The pooled prevalence of quality of life

**Fig. 2:**
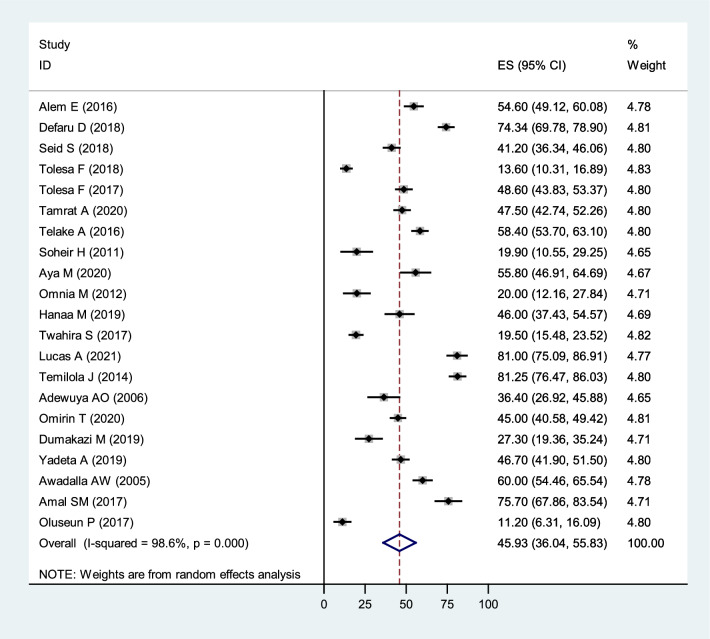
Forest plot of pooled prevalence of Quality of life among patients with mental illness in Africa 2022 (*n* = 21)

Twenty-one studies were included to estimate the pooled prevalence of poor QoL among African patients with mental illness. The prevalence of poor QoL in the included studies shows the minimum and maximum results of 11.2% [84] and 81.25% [77], respectively. The overall pooled prevalence of below mean QoL among patients with mental illness was 45.93% (36.04%, 55.83%; *I*^2^ = 98.6%, *p* < 0.001; Fig. 2). This result indicates that there was severe heterogeneity [60] between the studies (*I*^2^ = 98.6%, *p* < 0.001). The assumption of the random effects model, an estimate of random variation across studies/the Der Simonian and Laird’s pooled effect was applied. To reduce the random variations between the point estimates of the included studies, subgroup analysis and meta-analysis were carried out based on the type of mental illness, the measuring tool, the domains of QoL, and the Country.

### Subgroup analysis

A subgroup analysis by region (county), type of mental illness, measuring tool, and domains of QoL outcome variables was conducted because the included studies were highly heterogeneous.

### Based on a country

**Fig. 3:**
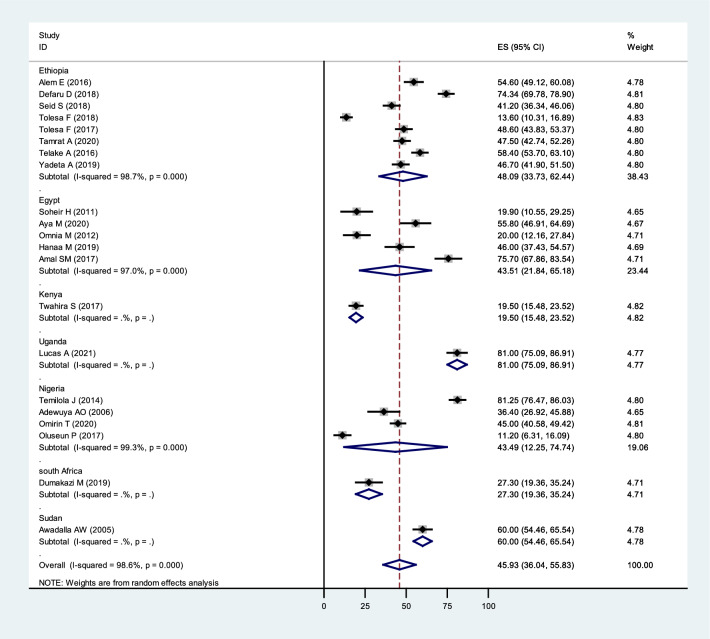
Subgroup analysis of the pooled prevalence of Quality of life among patients with mental illness in Africa based on the Country in 2022 (*n* = 21)

The meta-analysis revealed that the prevalence of poor QoL among patients with mental illness was high in Ethiopia (48.09%; 95% CI = 33.73,62.44), Egypt (43.51%; 95% CI = 21.84,65.18) and Nigeria (43.49%; 95% CI = 12.25,74.74). Single-study country prevalence was South Africa (27.30; 95% CI = 19.36,35.24), Sudan (60; 95% CI = 54.46,65.54), Uganda (81.00;95% CI = 75.09,86.91), and Kenya (19.50; 95% CI = 15.48,23.52), respectively (Fig. 3).

### Based on the type of illness

**Fig. 4:**
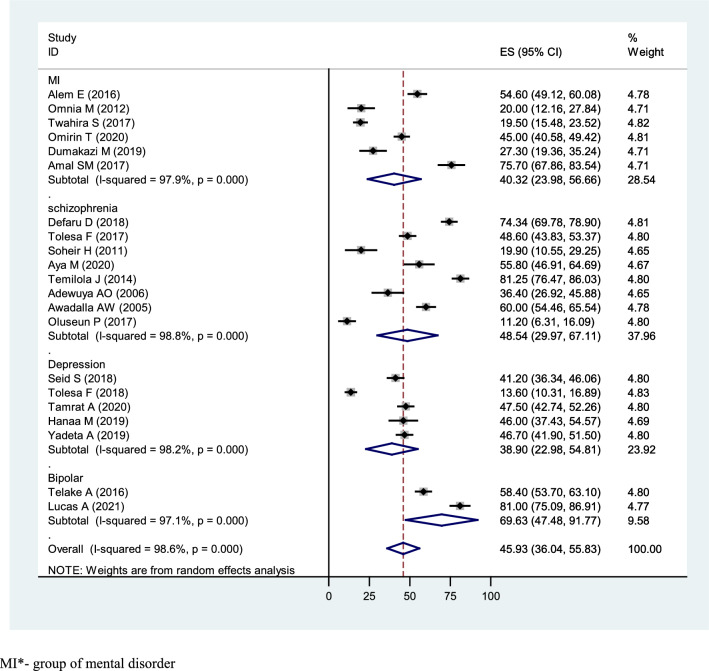
Subgroup analysis of the pooled prevalence of Quality of life among patients with mental illness in Africa based on the type of mental illness in 2022 (*n* = 21)

Subgroup analysis on the type of mental illness was done based on a pooled prevalence estimate. Our finding of literature has reported eight studies for diagnosis of schizophrenia, five studies for depression, two studies for bipolar disorder and six studies with a group of mental disorder diagnosis (MI). Poor QoL was high among bipolar patients with a prevalence of 69.63% (95% CI = 47.48,91.77), followed by schizophrenia (48.53%; 95% CI = 29.97,67.11), group of mental disorder (MI) (40.32%; 95% CI = 23.98,56.66), and depression (38.90%;95% CI = 22.98,54.81), respectively (Fig. 4).

### Based on the measuring tool of quality of life

**Fig. 5:**
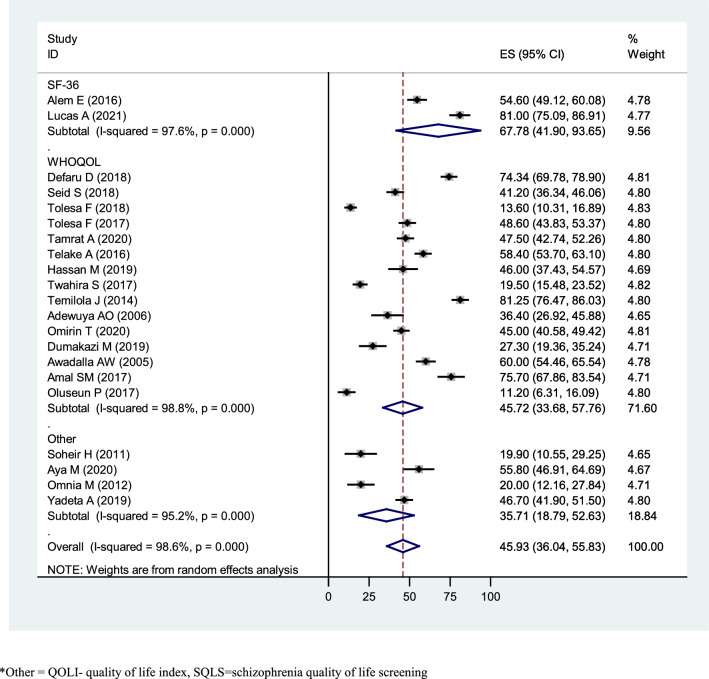
Subgroup analysis of the pooled prevalence of Quality of life among patients with mental illness in Africa based on the measuring tool of QoL in 2022 (*n* = 21)

Subgroup analysis was conducted based on the type of screening tool used to measure QoL. The mean QoL results was 67.78 for SF-36 (95% CI = 41.90, 93.65), 45.72 for the WHOQOL index (95% CI = 33.68, 57.76), and 35.71 for the other tools (QOLI, SQLS (Schizophrenia Quality of Life Scale) and EUROHIS) (95% CI = 18.79, 52.63) (Fig. 5).

### Based on domains of Quality of life

**Fig. 6:**
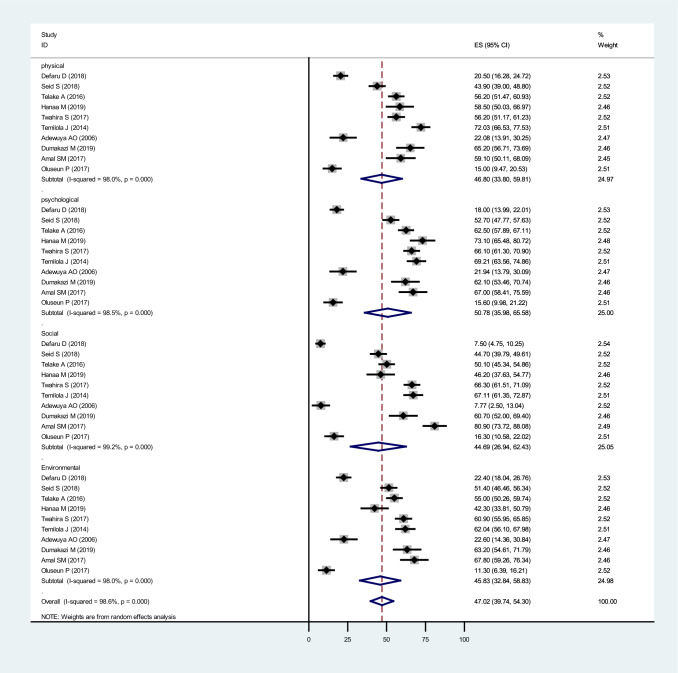
Subgroup analysis of the pooled prevalence of QoL among patients with mental illness in Africa based on domains of QoL in 2022 (*n* = 10)

Subgroup analysis on QoL domains shows a significant difference in each domain. Ten articles which addressed the domains of QoL with measuring tool WHOQOL brief. In our review pooled prevalence of mean QoL in psychological domains was 50.78% (95% CI = 35.98,65.58), 46.80% (95% CI = 33.80,59.81) for the physical domain, 45.83% (95% CI = 32.84,58.83) for the environmental domain, and 44.69% (95%CI = 26.94,62.43) for the social domain (Fig. 6).

### Meta-regression

To determine the cause of heterogeneity, we did a meta-regression analysis in addition to the subgroup analysis. A meta-regression analysis was performed using variables from the study participants, the number of participants, and the year of publication. The variables on meta-regression were not found to be significant sources of heterogeneity.

### Publication bias

**Fig. 7:**
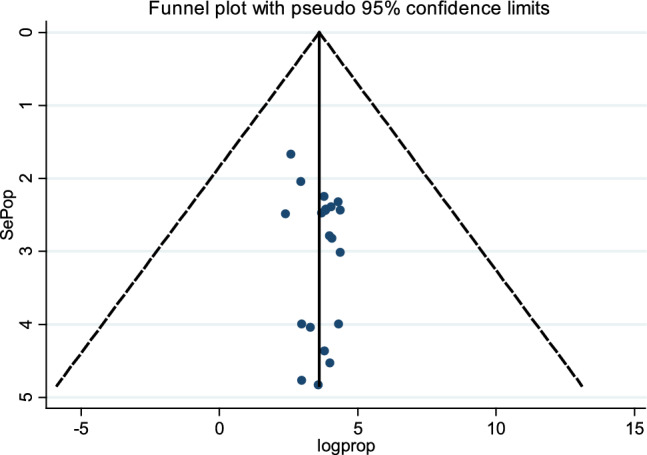
Funnel plot showing publication bias of prevalence of Quality of life, a systematic review, and meta-analysis, in Africa, 2022 (*n* = 21)

There were two techniques used to identify publication bias in systematic reviews and meta-analyses. When the funnel plot was examined, there was no indication of publication bias (Fig. 7). Additionally, Egger's weighted correlation was used to explore publication bias objectively. The test findings showed no publication bias (β = 0.22; standard error = 0.18; *P* = 0.24).

### Factors associated with poor quality of life among people with mental illness

**Fig. 8:**
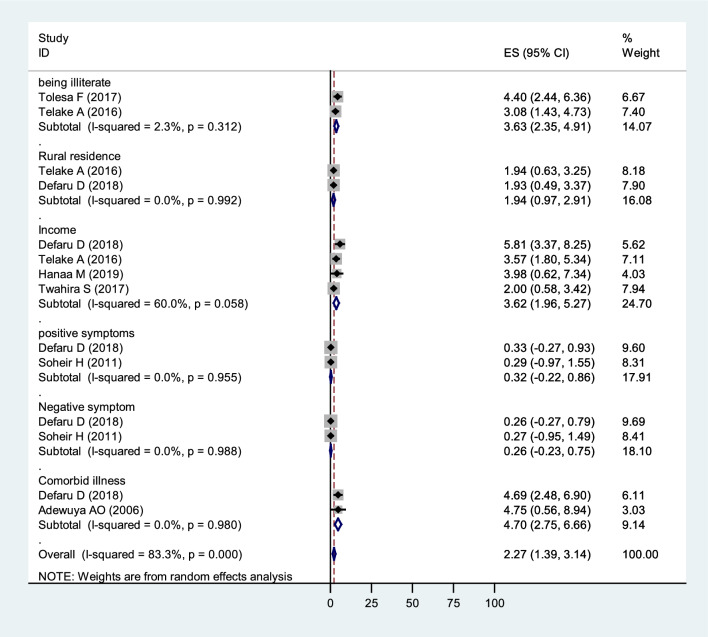
Factors associated with Quality of life among patients with mental illness, a systematic review, and meta-analysis, in Africa,2022 (*n* = 21)

In our synthesis, nine primary articles examined factors associated with QoL and found at least one factor associated with QoL. Among these were single marital status, unemployment, education to primary school, illiteracy, rural residence, having more than ten years of illness duration, poor social support, stigma, low monthly income, nonadherence to medication, positive symptoms, negative symptoms, psychopathology, tobacco use, khat use, comorbid physical illness, being on risperidone, and depression comorbid with other diseases. Specifically, two studies [70, 75] indicated that those unable to read and write had a lower QoL than those able to read and write. Additionally, two other studies [65, 70] reported that those living in rural areas had a lower QoL than those living in urban areas. Four studies [65, 70, 74, 75] showed that people with a low monthly income had a lower QoL than those with a higher monthly income. Two articles [65, 71] showed that patients with positive symptoms reported a poorer QoL than those who did not have positive symptoms and that patients with negative symptoms had poorer QoL than those patients who did not have negative symptoms. Two studies [65, 78] showed that having comorbid physical illness was associated with poorer QoL (Fig. 8).

## Discussion

This systematic review and meta-analysis aimed to establish a new estimate for poor QoL of patients with mental illness in Africa and to examine predictors of poor QoL. The review included 21 high-quality articles that measured the QoL among people with mental illness in Africa. The articles were separate studies that estimated the prevalence and associated factors of QoL among people with mental illness in seven African countries. No research from the remaining 47 African countries was identified. The included countries were Ethiopia, Egypt, Nigeria, Kenya, Sudan, South Africa, and Uganda, with an over-representation of articles from the northern African countries of Ethiopia and Egypt. This is important contextual information to note when considering the discussion of the findings. Given that notable intercultural differences may affect how mental illnesses are perceived in between and within countries, these findings should be interpreted with care, noting that they may not be projected as an absolute representation of Africa.

The results of this systematic review and meta-analysis suggest that poor QoL impacts many Africans with mental illness across the seven countries covered in the review. The findings have shown that the pooled prevalence of poor QoL among people with mental illness was 45.93% (95% CI = 36.04,55.83), indicating that poor QoL impacts half of adults with mental illness. The prevalence of poor QoL among patients with mental illnesses was lower than QoL reported among patients with other conditions, such as diabetic patients (66.5%) and cancer patients (58%) [85, 86], but like a systematic review and meta-analysis conducted to examine QoL in patients with Schizophrenia worldwide which was estimated at 40.66 (95% CI = 37.66, 43.66)[87]. These differences may be due to QoL variations among different illnesses.

Of the twenty-one articles reviewed from across primary studies, there was significant diversity and inconsistencies in the reported outcomes. For instance, the highest (81.25%) and the lowest (13.6%) prevalence of poor QoL were reported in Nigeria [77] and Ethiopia [67], respectively. These variations may reflect differences in measuring instruments for QoL and/or differences in the impacts of mental illness on the QoL of participants.

The prevalence of poor QoL was different in subgroup analysis based on the diagnosis. The current study found higher rates among patients diagnosed with bipolar disorder 69.63%, followed by Schizophrenia, 48.53%, and depression 38.90%. Other studies have shown that participants with bipolar disorder had poorer QoL than the other groups with different mental health conditions [88]. The poorer QoL of those with bipolar disorder may relate to more “anti-social” behaviours associated with the illness that could impinge on QoL [89, 90]. Other studies comparing QoL between mental health conditions have found varying results. For example, a single study conducted on patients in Singapore with Schizophrenia and depressive disorder revealed that patients with Schizophrenia had better QoL when compared with patients with major depressive disorder [91]. The lowest QoL was found among individuals with unipolar depression [33]. These differences in QoL could have been a result of the small number of studies on bipolar disorder that were included in the review here or potentially variations in the country context.

Our systematic review prevalence rates are also varied by QoL domain. Findings showed a higher prevalence of poor QoL in the psychological domain at 50.78%. The physical domain was 46.80%, environmental 45.83%, and social 44.69%. This result is like a population-based cohort study in Iran that shows a high prevalence of psychological domains: psychological (48%), physical (43%), environment (39%), and social relation (38%) [92]. The findings of earlier studies conducted in other countries, including Brazil, South Africa, and Germany, support these findings [42, 93, 94]. This suggests that of the underlying mental health QoL dimensions, the psychological domain of QoL is most affected by mental illness, followed by the domains of physical, environmental, and social relations.

This systematic review and meta-analysis identified pooled effect estimate predictors of the QoL of people with mental illness. Our study found that as the mean pooled effect estimate score of positive symptoms increased by one unit, the total QoL score decreased by 0.32 (p < 0.82). This outcome was consistent with numerous findings from earlier research [95–97]. Positive symptoms had the most significant negative relationship with health-related QoL [96]. Patients with negative symptoms, on the other hand, increased their pooled effect estimate by one unit, while their total QoL score decreased by 0.26 (*p* < 0.9). These findings further support the idea that patients exhibiting negative symptoms may retreat from their surroundings, show little interest in routine social interactions, and appear emotionless and flat [98]. Their QoL seems to be more affected by negative symptoms. Improvements in QoL are strongly correlated with improvements in negative symptoms [99, 100]. These findings suggest that using detailed intervention strategies to manage symptoms is essential to improving the QoL of people with mental illness.

Individuals with a low income were 3.62 (OR = 3.62, CI 1.96, 5.27) times more likely to have a poor QoL than those with a high income, according to pooled effect estimates from four studies [65, 70, 74, 75]. This association is consistent with studies conducted in Hong Kong on poverty and health-related QoL of adults from low-income households [101] and in the United States with people with low income [102]. Our analysis found that, despite gaps in the evidence, income likely has an impact on QoL [103], where monthly income and QoL were significantly associated [95]. This may be the case because many people with mental illnesses cannot maintain employment [104].

In this review, two studies in Ethiopia found that being illiterate is associated with the QoL of patients with mental illness. Participants without literacy skills were more likely to have a low QoL than participants with a degree or higher qualifications [68, 75]. This is similar to a study with people with Schizophrenia and QoL in Jima Ethiopia that indicates a negative correlation between the physical health domain and lack of formal education [100]. Each study's regression result showed that QoL was negatively affected by educational status. In our systematic review and meta-analysis, the pooled effect estimates of academic level showed that being illiterate was associated with being 3.63 times more likely to have a poor QoL than being educated to high school and above (OR = 3.63; CI 2.35, 4.91).

Several included studies found that patients with mental illnesses who lived in rural areas had a lower QoL than those who lived in urban areas. Two studies in Ethiopia found that living in a rural residence was associated with being 1.94 and 1.93 [70, 100] times more likely to have poor QoL. These differences could be because rural dwellers with mental illness are more likely to have more inadequate access to health and support services than urban dwellers, which may affect their QoL [105]. However, the pooled effect estimates in this meta-analysis revealed that rural residency had no association with poor QoL (OR = 1.94; CI 0.97,2.91). The reason for the pooled estimate to be non-significant could potentially be a false-significant finding in one of the included primary studies.

Included studies showed an association between comorbidity problems and patient related QoL. For example, a cross-sectional survey undertaken in Ethiopia among patients with Schizophrenia attending follow-up treatment in an outpatient clinic showed comorbidities were associated with a 4.69 great chance of reporting poor QoL [65]. Another study conducted in Nigeria on subjective QoL showed that people with comorbidities were 4.75 times more likely to have poor QoL [78]. The pooled effect estimates of having comorbid physical illness was associated with poor QoL in the meta-analysis, with OR: 4.7 (2.75, 6.66). These comorbidities could be physical issues that impact the signs and symptoms of mental illness, raise healthcare costs, and affect patients' attitudes toward treatment and QoL.

In terms of other predictors of QoL, one study in Nigeria showed that those who were unemployed were 3.75 times more likely to have poor QoL than those who were employed [78]. This may be because work can normalize an individual's perception of their value, whereas unemployment may reduce it. The same study found that people with Schizophrenia who were employed had a significantly higher health-related QoL than those who were not employed [106]. Marital status was another sociodemographic factor associated with respondents' QoL; being married was 2.96 times less likely to develop poor QoL than being divorced or widowed in a study in Kenya [107]. This is supported by a study done in Nigeria among patients with major depressive disorder, which found that married patients reported having better QoL than single patients [108].

Duration of illness was also identified as a predictor for QoL for people with mental illness. In a study conducted in Ethiopia, patients who had been ill for ten years or more were about three times more likely to have a poor QoL than those who had been sick for less than ten years [70]. However, the finding in this study was not supported by other studies from other countries.

Social support was among the factors associated with QoL. In a Nigerian study, for example, social support was found to be a predictor of QoL. Patients with poor social support were reported to be 4.6 times more likely to have poorer QoL than patients with stronger social support [78]. Social support was also found in an Ethiopian study with patients with severe mental illness reported to be adversely correlated with all categories of QoL except the physical domain [109]. Other studies have found a similar link between social support and QoL, including in patients with Schizophrenia and bipolar disorders in India [110], among psychiatry patients in Egypt [83], and patients with anxiety in Cyprus [111].

Another predictor of QoL was stigma. An Ethiopian study found that an increase of self-stigma by one unit in patients with mental illness decreased the QoL by 4.1% [64]. Evidence exists that stigma can impact a person's condition and affect patients' ability to access suitable and qualified medical care [112, 113].

Medication nonadherence is another factor that predicts the QoL of patients with mental illness. Defaru and colleagues [100] discovered that patients with drug nonadherent were 5.81 times more likely to develop poor QoL than adherent patients. Furthermore, published studies have identified general psychopathology as more strongly related to poor QoL among outpatients with mental illness. In this review, we identified a study that showed that as general psychopathology symptoms increased by a unit, patients’ QoL decreased by 0.22 units [100]. This finding is supported by previous systematic reviews and meta-analyses of the global context of schizophrenia patients [96].

Finally, we found other factors that predicted the QoL of patients with mental illness in a single study but could not be pooled. These factors give further clues about potential factors associated with QoL for people with mental illness: These factors included substance use [100], being on risperidone medication [68], having comorbid depression [68], having sexual dysfunction[68], having 2–4 episodes of symptoms per year [70], taking antipsychotic and antidepressants [70], being not functional [74], having psychotic symptoms[76], having suicidal symptoms[76] and having comorbid anxiety and depressive symptoms[78].

Although the prevalence of poor QoL differs in various countries, as stated above, more information still needs to be on this distinction. The reason for variations could be multifactorial. For example, nations like Ethiopia may need a robust health system supporting mental illness patients. Still, they have high social cohesion and collectivist lifestyles, which help patients with mental illnesses, cushioning the impact of these illnesses. Countries like South Korea, Taiwan, Denmark, and Australia may have better access to robust healthcare systems for patients with shorter illness durations [114]. As a result of these variations, the QoL may be contextually different across the countries. Evidence in Europe shows mental illness prevalence to be different by geographical location [18–20]. The impact of sociodemographic and clinical factors may also vary between country contexts. For example, the effect of unemployment on QoL for someone living with mental illness in a country with a robust welfare system may be quite different for someone with no unemployment welfare payments. Likewise, country variations in cultural understandings around gender may affect the impact of this on QoL. These findings highlight the importance of considering country contexts when planning policy and practice responses to the QoL impacts of mental illness.

## Strengths and limitations

This systematic review and meta-analysis examined some predictor variables for QoL and showed pooled prevalence in domains of QoL. The included studies used a set of diagnostic tools that consistently measure the outcome QoL variables, and this enabled us to generate objective estimates of the overall prevalence of QoL among patients with mental illness in Africa. The current study is the first as a systematic review and meta-analysis in African countries examining the QoL of people with mental illness. However, there are some limitations. As noted, the included articles were from seven African countries, representing fewer than a quarter of all African countries. Countries that were represented could have more active research activities than their counterparts, leading to more publications in the subject matter. It is also theorized that other issues, such as mental health stigma and mental health literacy, could deter research activities in countries where the articles could not be found.

Given that there are likely variations in the contextualisation of mental illness and the impacts on QoL between countries, this is a significant limitation to consider. Second, some of the articles included in the current systematic review and meta-analysis had small sample sizes, including articles by Soheir H 2011[71], Aya M 2020[72], and Omnia M, 2012[73], too low to be generalized for a patient with mental illness and the tool that used to measure QoL is not used in other African countries other than Egypt. Third, some of the included studies reported beta coefficients through regression or evaluated general QoL, so they were excluded from the final analysis of determinant factors of QoL. Still, we had them in the prevalence study. Fourth, there needed to be more studies to do pooled effect estimates for all variables associated with poor QoL, and some had only two studies contributing to the pooled effect estimate. Finally, due to the limitations of previous systematic reviews and meta-analyses on this topic, it was only sometimes possible to directly compare our findings, so we have discussed QoL for other conditions as a comparator. The results of this study should consider these limitations.

## Conclusion and recommendation

Our systematic review and meta-analysis indicate that poor QoL among African patients with mental illness is a significant public health problem. Almost half of the patients with mental illness in the included African studies have poor QoL. Our findings also identified factors associated with poor QoL, including having a low monthly income, having positive symptoms, having negative symptoms, being illiterate, and having a comorbid medical illness. These factors give some clues as to pathways through which mental illness impacts QoL, and therefore, potential intervention opportunities for programs seeking to improve QoL among people with mental illness.

## Data Availability

This published article and its supplementary information files include all data generated or analysed during this study.

## Abbreviations

AOR: Adjusted odds ratio
COR: Crude odds ratio
CI: Confidence interval
MOOSE: Meta-analysis of observational studies
OR: Odds
QoL: Quality of life
SPSS: Statistical package for social science
WHO: World health organization
